# Identification of a novel subtype of SPP1 + macrophages expressing SIRPα: implications for tumor immune evasion and treatment response prediction

**DOI:** 10.1186/s40164-024-00587-3

**Published:** 2024-12-18

**Authors:** Kun Chen, Yida Li, Jianjiao Ni, Xi Yang, Yue Zhou, Yechun Pang, Ruiting Ye, Hongru Chen, Silai Yu, Peng Wang, Zhengfei Zhu

**Affiliations:** 1 Department of Radiation Oncology, Fudan University Shanghai Cancer Center, 270 Dong An Road, Shanghai, 200032 China; 2 Department of Oncology, Shanghai Medical College, Fudan University, Shanghai, China; 3 Shanghai Clinical Research Center for Radiation Oncology, Shanghai, China; 4 Shanghai Key Laboratory of Radiation Oncology, Shanghai, China; 5 Department of Integrative Oncology, Fudan University Shanghai Cancer Center, Shanghai, China; 6 The Affiliated Suzhou Hospital of Nanjing Medical University, Suzhou Municipal Hospital, Gusu School, Nanjing Medical University, Suzhou, Jiangsu China; 7 Department of Hepatic Oncology, Zhongshan Hospital, Fudan University, Shanghai, China; 8 Liver Cancer Institute, Zhongshan Hospital, Fudan University, Shanghai, China; 9 Institute of Thoracic Oncology, Fudan University, Shanghai, China

**Keywords:** Esophageal squamous cell carcinoma, SPP1 + macrophages, SIRPα, Tumor microenvironment, Immunotherapy

## Abstract

**Background:**

SPP1 + macrophages are among the major phagocytic cells, yet promoting tumor immune evasion and predicting unfavorable prognosis, in various cancer types. Meanwhile, the predictive value of the abundance of SPP1 + macrophages in patients receiving immunotherapy remains debatable, indicating the potential existence of subtypes of SPP1 + macrophages with diverse biological functions.

**Methods:**

The single cell RNA sequencing data of myeloid cells integrated from several cancers including esophageal squamous cell carcinoma was analyzed for characterizing the function and cellular interactions of SPP1 + macrophages expressing SIRPα. Multiplexed immunohistochemistry was used to quantify the quantity and spatial distribution of SPP1 + macrophages expressing SIRPα. Kaplan–Meier method was used for survival analysis. In vitro and in vivo studies investigating the function of SPP1 + macrophages were performed.

**Results:**

SPP1 + macrophages possessed a high phagocytic signature and could engulf more tumor cells in vitro and in vivo. SIRPα expression could represent the phagocytic activity of SPP1 + macrophages and delineated subsets of SPP1 + macrophages with different functions. SPP1 + SIRPα + macrophages showed close spatial distance to tumor cells and positively correlated with PD1 + CD8 + T cells. A high abundance of SPP1 + SIRPα + macrophages at baseline corresponded to patients’ response to PD-1/PD-L1 inhibitors.

**Conclusion:**

A novel subtype of SPP1 + macrophages expressing SIRPα was identified and their abundance predicted patients’ response to PD-1/PD-L1 inhibitors.

**Supplementary Information:**

The online version contains supplementary material available at 10.1186/s40164-024-00587-3.

## Background

Macrophages are CD68 + leukocytes that play a crucial role in innate immunity and are proficient in triggering adaptive immune responses [1–3]. The phagocytosis of tumor cells mediated by macrophages is the bridge between innate and adaptive immunity [4]. Usually, proinflammatory M1 macrophages are considered capable of phagocytosing tumor cells [5]. Recently, SPP1 + macrophages, characterized by high levels of *APOE*, *SPP1, TREM2,* and *MMP9* that were initially identified in adipose tissue as lipid-associated macrophages, were reported to be enriched in hypoxic and necrotic tumor regions and serve as the phagocytic cells for phagocytosing dying cells in colon cancer [6, 7]. Meanwhile, SPP1 + macrophages were also reported to contribute to tumor progression by prompting the invasiveness of tumor cells or T cell suppression, and the enrichment of SPP1 + macrophages was reported to predict unfavorable prognosis across multiple cancer types [8–11]. Therefore, the exact biological roles of SPP1 + macrophages in tumor microenvironment (TME) warrants further exploration. In addition, the effect of SPP1 + macrophages on the efficacy of immunotherapy remains controversial. In non-small cell lung cancer (NSCLC), a high lung cancer activation module score, which is determined by activated T cells, IgG^+^ plasma cells, and SPP1^+^ macrophages, correlated with enhanced response to immunotherapy [12]. Conversely, hepatocellular carcinoma (HCC) patients with high SPP1 expression were related to less therapeutic benefit from anti-PD-L1 therapy [11]. The controversial characteristics of SPP1 + macrophages in immunotherapy suggest the existence of subtypes of SPP1 + macrophages with diverse biological functions and distinct predictive abilities.

SIRPα is a transmembrane molecule that is mainly expressed by phagocytes, such as macrophages, DCs, and neutrophils, especially in macrophages. When bound with its ligand CD47, the inhibitory signal of phagocytosis is delivered to phagocytes [13]. Clinical evidence has revealed that CD47-SIRPα interaction in tumor-associated macrophages is a pro-tumorigenic factor and predictor of survival [14]. However, the expression profile of SIRPα in myeloid cells and the effect of its expression on the phenotype of myeloid cells has not been fully studied.

Esophageal carcinoma is the seventh most common cancer and ranks the sixth among cancer-related deaths worldwide, with esophageal squamous cell carcinoma (ESCC) being the predominant histological subtype in China [15, 16]. Because of the difficulty in early diagnosis and the lack of efficient treatments, ESCC has a poor prognosis with 5-year survival rates of approximately 20%–30% [17]. Though SPP1 + macrophages were also identified in ESCC tissues through single-cell RNA sequencing (scRNA-seq), their biological roles in ESCC remained elusive [18, 19]. Recently, PD-1/PD-L1 inhibitors have been applied in ESCC and moderately improved the overall survival of patients [19, 20]. However, a considerable percentage of patients fail to benefit from such therapy. Therefore, an improved, predictive biomarker-based patient stratification criterion may be useful to design multimodal immunotherapy regimens.

In this study, we explored the phagocytic capacity of myeloid cells by using published scRNA-seq datasets from ESCC and other cancer types including HCC, colon adenocarcinoma (COAD), NSCLC, stomach adenocarcinoma (STAD), and pancreatic ductal adenocarcinoma (PDAC). We found that SPP1 + macrophage exhibited higher phagocytic ability than other myeloid sub-clusters. Within SPP1 + macrophages, SIRPα positive cells had higher antigen presentation ability, lysosome and phagosome activity, and immune-suppressive activity compared with SIRPα negative ones. Moreover, we demonstrated that the baseline abundance of SPP1 + SIRPα + macrophages predicted favorable clinical outcomes in patients receiving immunotherapy.

## Methods

### Data collection

scRNA-seq data of ESCC, HCC, NSCLC, COAD, STAD, and PDAC were downloaded from GEO (GSE145370, GSE154763, GSE160269, GSE164522, GSE202462, GSE148071, GSE205013 and GSE206785). The scRNA-seq data from various cancer types were integrated, and data on myeloid cells were subjected to analysis. RNA-seq samples of NSCLC patients (n = 27) who received immunotherapy were downloaded from GSE135222 [21]. RNA-seq data of metastatic urothelial carcinoma (mUC) patients (n = 255) treated with atezolizumab was obtained from IMvigor210 [22]. RNA-seq data of HCC patients who received atezolizumab plus bevacizumab (n = 253) at baseline was obtained from IMbrave150 and GO30140 clinical datasets [23–25]. 206 melanoma patients who received pembrolizumab or nivolumab treatment, among which 121 melanoma patients with RNA-seq data were enrolled [26]. RNA-seq data of ESCC and HCC patients were downloaded from The Cancer Genome Atlas Program (TCGA). To validate the phagocytic ability of SPP1 + macrophages in vivo, we reanalyzed the scRNA-seq data from a mouse lung cancer model (GSE217368) [27].

### Patient samples

ESCC cohort 1 consists of 240 treatment-naïve, histologically confirmed ESCC patients who underwent primary surgical resection at Fudan University Shanghai Cancer Center, Sun Yet-Sen University Cancer Center, and Nantong Tumor Hospital in 2013 (Table S1, Additional file 1). ESCC cohort 2 consisted of 18 patients who received neoadjuvant chemoimmunotherapy and following surgical resection at Fudan University Shanghai Cancer Center (Table S3, Additional file 1). The HCC tissue microarray (TMA), the COAD TMA, the NSCLC TMA, and the STAD TMA were generated from samples of patients from Changsha Yaxiang Biotechnology Co.LTD. and approved by the Life Sciences Ethics Committee of Changsha Yaxiang Biotechnology Co., LTD (Table S2, Additional file 1).

### Tissue processing for TMA construction

For the multiplex immunohistochemistry (mIHC) cohort, archival tissue blocks of samples of ESCC patients were obtained and confirmed as ESCC by the pathology department of the centers listed above. The tonsil and lymph nodes were used as positive staining controls. Briefly, two different formalin-fixed paraffin embedded blocks of tumor tissues from each patient were obtained and spotted in adjacent 2.5 mm cores to capture tumor heterogeneity. Each block was reviewed and the region of interest was marked by pathologists after hematoxylin and eosin staining. A recipient paraffin block was made with a duplicate 2.5 mm punch from tissue samples using Tissue Arrayer following the positional blueprint. Next, the recipient array blocks were incubated at 53 °C for 2 h to adhere cores in the recipient block. After cooling, the blocks were sectioned with a microtome (3 µm thick) and fixed on the charged slides. The TMA was finely layered with paraffin to prevent antigen decay and stored at 4 °C until use.

### scRNA-seq data processing

The Seurat package (version 4.4.0) was used for quality control and subsequent analysis. For quality control, cells with red blood cell genes or mitochondrial RNA content > 10% were filtered out. Cells that had gene counts < 200 or gene counts > 3000 were removed. Highly variable genes were selected with the FindVariableGenes function and subjected to subsequent Normalization and ScaleData function. The scale data then underwent principal component analysis with RunPCA and the top 30 principal components (PCs) were used to perform unsupervised clustering with FindClusters. The resolution parameter (Res) set as 0.3 and k for k-nearest neighbor algorithm was 30 for clustering analysis. The marker genes of specific clusters were identified with the FindAllMarkers function. The cluster was annotated with the marker genes. To merge myeloid cells of various cancers, we ran the Seurat integration procedure or a harmony analysis for batch correction using the Runharmony function with harmony packages (version 1.1.0). The clustering results were visualized on the UMAP plots generated with RunUMAP function.

Annotation of cell clusters was based on the expression of known genes. *CD68*, *LYZ*, *C1QC*, and *FOLR2* for macrophages; *TPSAB1*, *TPSB2*, and *CPA3* for mast cells; *CD1C*, *CLEC9A*, *LAMP3*, and *CLEC10A* for dendritic cells; *CD14*, *S100A8*, *S100A9*, and *LST* for monocytes; *FCGR3B* and *CXCL8* for neutrophil cells [28].

### Analysis of gene sets signature score

Gene set signature score in the scRNA-seq data was calculated with the AddModuleScore function of the Seurat package (version 4.4.0). The phagosome, lysosome, antigen presenting, and immune inhibiting gene expression signatures are supplied in Table S5, Additional file 1. The phagosome, lysosome, and antigen presenting signaling pathways were downloaded from the Kyoto Encyclopedia of Genes and Genomes (KEGG). The top 30 unregulated feature genes were defined as SPP1 + SIRPα + macrophages signature. The T cell suppressed signature of macrophages was derived from published studies [29]. After the calculations, each cell was assigned a corresponding gene set score. For the bulk RNA-seq data, Gene Set Variation Analysis (GSVA) (version 1.48.3) was used to calculate the cell specific gene signature score to represent the abundance of the specific cell type.

### Gene set enrichment analysis (GSEA)

To obtain the enriched signaling pathway in SPP1 + SIRPα + macrophages, the differently expressed genes in SPP1 + SIRPα + macrophages were first calculated and ranked by the fold change. The hallmark gene sets utilized were downloaded from the Human Molecular Signatures Database. Then the GSEA results were calculated by clusterProfiler (version 4.8.3) and presented by enrichplot (version 1.20.3).

### Phagocytosis assays

To investigate the phagocytic ability of SPP1 + macrophages in vitro. We first obtained SPP1 + macrophages by co-culturing THP-1 macrophages with the ESCC cell line KYSE150 in an indirect manner using a 0.7 µm Transwell chamber for 48 h [9, 30]. In brief, THP-1 cells were first treated with Phorbol 12-myristate 13-acetate (PMA) for 48 h to differentiate into THP-1 macrophages. Then THP-1 macrophages and the ESCC cell line KYSE150 were co-cultured indirectly using a 0.7 µm Transwell chamber for another 48 h to get SPP1 + macrophages-like tumor macrophages. We then labeled the tumor cells with CFSE and co-cultured the tumor cells with THP-1-derived SPP1 + macrophages or control THP-1 macrophages for 4 h within serum-free medium. CD11b + CFSE + cells were regarded as SPP1 + macrophage that engulfed tumor cells**.**

### Quantitative real-time PCR assay

Total RNA was extracted from cultured cells with TRIzol (Thermo Fisher Scientific, 15596026CN) and reverse transcribed. Aliquots of cDNA were amplified with SYBR Green PCR master mix on a IQ5 Real-Time PCR system (Bio-Rad) using the primers shown in Table S4, Additional file 1. GAPDH was amplified in parallel to normalize expression of the genes of interest.

### Cell–cell interaction analysis

To investigate the cell interactions between SPP1 + SIRPα + macrophages with malignant epithelial cells and CD8 + T cells, we conducted analysis with CellChat (version 1.6.1) to explore the specific cell interactions. The number of interactions and interaction strength between SPP1 + SIRPα + macrophages with malignant epithelial cells and CD8 + T cells was exhibited by Circle plot. Significant ligand-receptor pairs, which contribute to the signaling from macrophages subsets to epithelial cells and CD8 + T cells was presented by Bubble plot. *p*-values are calculated by one-sided permutation test.

### Survival analysis

For survival analyses or progression-free survival analyses of samples of patients who received immunotherapy stratified by SPP1 + SIRPα + macrophages, the signature scores were first calculated by GSVA (version 1.42.0) or the ratio of SPP1 + SIRPα + macrophages was calculated. Samples were dichotomized using the median value or best cut-off point determined by “surv_cutpoint” function of survminer package (version 0.4.9). The Kaplan–Meier method was used to obtain survival curves, and *p*-values were calculated using a log-rank test unless otherwise specified. The analyses were conducted by R package survival (version 3.5.8).

### Multiplexed immunohistochemistry and spatial analysis

The TMA first underwent deparaffinization and dewaxing. Antigen retrieval was performed using a AR6 buffer (pH 6.0, PerkinElmer) in a pressure cooker. After cooling, slides were washed and blocked with freshly prepared 3%H_2_O_2_. To remove endogenous peroxidase, a blocking solution containing 10% normal goat serum (S-1000, Vector Labs, Burlingame) was applied. Sections were then incubated with monoclonal antibodies against antigens including SIRPα (Origene; TA381503), CD47 (Abcam; ab226837), CK (Abcam; ab80826), CD68 (Maxim; clone KP1), PD-1 (Abcam; ab237728), and CD8 (Abcam; ab101500) followed by incubation with corresponding isotype-matched polymer-based horseradish peroxidase-conjugated secondary antibodies (Vector Laboratories).

After washing, a tyramide signal amplification step was performed to mediate the horseradish peroxidase–covalent binding of different fluorophores. In tyramide signal amplification–based multiplexing, a subsequent cycle of antigen retrieval steps was performed after each round of Opal to remove unbound antibody-dye complexes of the previous staining. Tissues were then counterstained with DAPI (D9542, Sigma-Aldrich), mounted in Vectashield Hardset^**®**^ fluorescence mounting medium (Vector Labs, Burlingame), imaged using the Vectra3.0 Multispectral Imaging System and analyzed using inForm (version 2.4.0).

For spatial analysis, each cell was first classified according to the expression status of cell markers used in in-situ mIHC staining. The distance between every cell and every other cell in every TMA core was calculated using QuPath (version 0.5.1). The closest distance of any SPP1 + SIRPα + macrophages, SPP1 + SIRPα- macrophages, and SPP1 + macrophages to the panCK + tumor cells or CD8 + T cells was obtained.

### Statistical analyses

The abundance of cell types was determined by GSVA score, and the survival and progression-free survival were dichotomized using the median value or best cut-off point determined by the “surv_cutpoint” function of survminer package (version 0.4.9) unless otherwise stated. The Kaplan–Meier survival curves and progression-free survival curves were assessed using log-rank tests. Student’s *t*-test or Wilcoxon test was used to compare two groups. Statistical analysis was performed using R (version 4.4.0). A *p*-value < 0.05 was considered statistically significant.

## Results

### High phagocytic activity and SIRPα expression of SPP1 + macrophages in ESCC

**Fig. 1:**
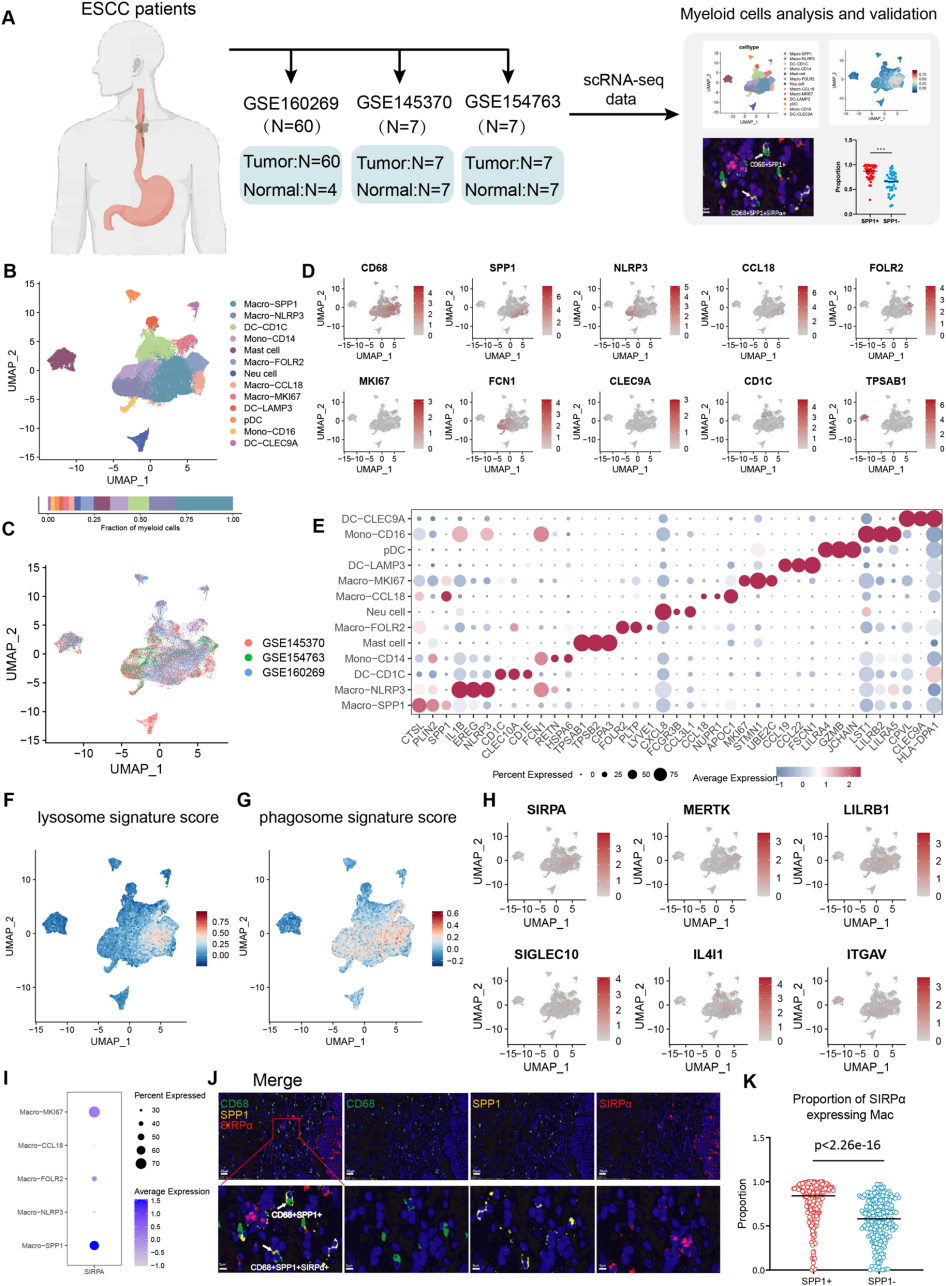
High phagocytic activity and SIRPα expression of SPP1 + macrophages in ESCC. **A** The acquisition and process procedure of ESCC scRNA-seq data. **B** UMAP of the myeloid cells towards various cell types in ESCC. **C** UMAP of the myeloid cells from various scRNA-seq data sources. **D** UMAP of the feature genes of various myeloid cell sub-clusters in ESCC. **E** The expression profile of the top differently expressed feature genes of the myeloid cell sub-clusters. **F** UMAP of the lysosome gene sets score in myeloid cells of ESCC. **G** UMAP of the phagosome gene sets score in myeloid cells of ESCC. **H** UMAP of expression of phagocytosis-related molecules in myeloid cells. **I** The expression profile of *SIRPA* across macrophage subsets in ESCC. **J** Representative in situ mIHC staining of CD68, SPP1, and SIRPα in ESCC samples (n = 240). **K** The proportion of SIRPα-expressing macrophages in SPP1 + macrophages and SPP1- macrophages in ESCC tumor tissues (n = 240). Statistical significance was assessed using the two-sided Wilcoxon test

To investigate the role of phagocytes in the TME of ESCC, we integrated the myeloid cells from 92 scRNA-seq samples (74 tumor samples and 18 normal tissues) across three published ESCC datasets (Fig. 1A). A total of 52,666 myeloid cells were identified and divided into 13 sub-clusters (Fig. 1B and C). Macro-SPP1 was characterized by high levels of *SPP1, CTSD, CTSB* and *TREM2*, suggesting it represents lipid-associated macrophages (Fig. 1D and E). Macro-MKI67 highly expressed proliferation-related genes, such as *MKI67* and *STMN1* genes, suggesting its proliferative state. Notably, Macro-MKI67 also selectively expressed feature genes of Macro-SPP1; however, it remains elusive whether Macro-MKI67 represents a transition state of Macro-SPP1. Macro-NLRP3 highly expressed inflammation-related genes, such as *EREG, IL1B,* and *NLRP3.* Macro-FOLR2 highly expressed *FOLR2*, *PLTP*, and *LYVE1* genes, indicating it as the tissue-resident macrophage [31]. Macro-CCL18 highly expressed *CCL18* and *APOC1*, which high *CCL18* expression indicating an adverse role in the TME [32, 33]. Notably, Macro-CCL18 also expressed several feature genes of Macro-SPP1. The relationship between them remained further investigation. Monocytes were identified as Mono-CD14 (*S100A8, S100A9*, and *FCN1*) and Mono-CD16 (*LST1* and *LILRB2*). Dendritic cells (DCs) were separated into DC-CD1C (*CD1C, CLEC10A*, and *CD1E*), DC-LAMP3 (*CCL19, CCL22*, and *FSCN1*), DC-CLEC9A (*CLEC9A* and *HLA-DPA1*) and pDC (*LILRA4, GZMB*, and *JCHAIN*). Mast cells were characterized by high expression levels of *TPSAB1, TPSB2*, and *CPA3*, while neutrophils highly expressed *IFITM2, FCGR3B,* and *CXCL8.* The feature genes of the myeloid cell sub-clusters are shown in Table S6, Additional file 2.

To evaluate the phagocytic ability among the various myeloid sub-clusters, the phagosome and lysosome gene sets scores were calculated through the AddModuleScore function. Macrophages exhibited more powerful phagocytic ability compared with DC cells and monocytes (Fig. 1F and G). Previous studies have suggested that tumor cells play a vital role in the formation of SPP1 + macrophages [9, 30]. In our data, SPP1 + macrophages were primarily enriched in tumor tissues (Fig. S1A). Hence, to verify the phagocytic ability of SPP1 + macrophages, we co-cultured THP-1 macrophages with the ESCC cell line KYSE150 in an indirect manner to obtain SPP1 + macrophages -like tumor macrophages (Fig. S1B). After 48 h of co-culture, the expression levels of feature genes of SPP1 + macrophages, such as SPP1, TREM2, APOE, and GPNMB genes, all increased (Fig. S1C). Then tumor cells labeled with CFSE were co-cultured with THP-1-derived SPP1 + macrophages for 4 h in serum-free medium. The results showed that SPP1 + macrophages exhibited higher phagocytic ability compared with THP-1-derived macrophages (Fig. S1D-F).

Interestingly, we found that, in contrast to phagocytosis-related molecules such as MERTK, LILRB1, SIGLEC10, IL4I1, and ITGAV [6, 27, 34, 35], only the expression pattern of *SIRPA* in myeloid cells was consistent with the phagosome and lysosome scores distribution (Fig. 1H). Moreover, Macro-SPP1 exhibited high expression of *SIRPA* compared with other macrophages in ESCC (Fig. 1I). In-situ mIHC staining using antibodies against CD68, SIRPα, and SPP1 in our in-house ESCC cohort 1 patients identified macrophages co-expressing SPP1 and SIRPα and SPP1 + macrophages possessed a high proportion of SIRPα expression (Fig. 1J, K and Table S1). These results showed the existence of SPP1 + macrophages with high phagocytic activity within ESCC and characterized by high SIRPα expression.

### Vigorous phagocytic activity of SPP1 + macrophages is prevalent across multiple cancer types

**Fig. 2:**
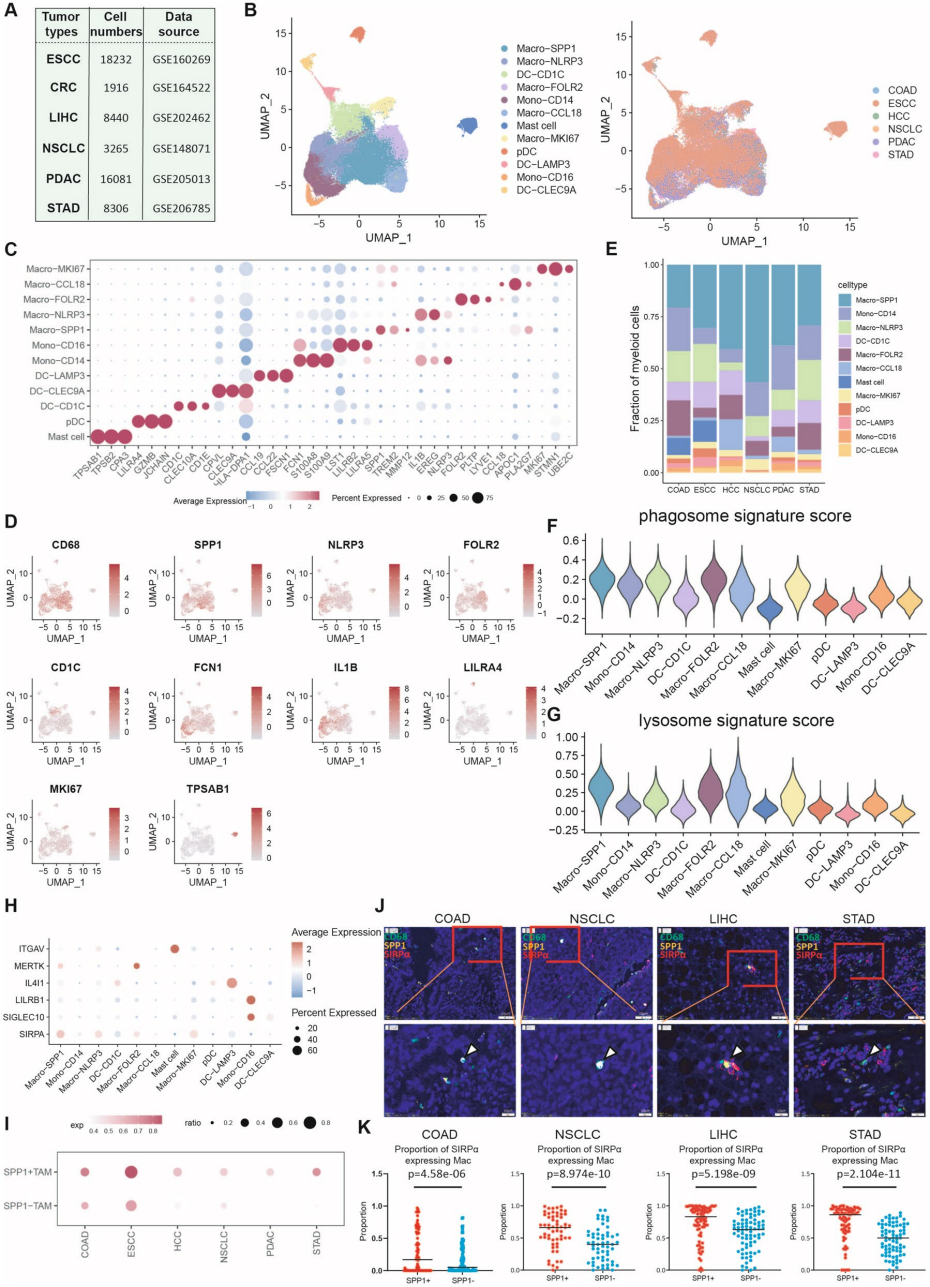
Vigorous phagocytic activity of SPP1 + macrophages is prevalent across multiple cancer types. **A** scRNA-seq data sources. **B** UMAP of the myeloid cells towards various cell types (left) and in different cancer types (right). **C** The expression profile of the top differently expressed feature genes of the myeloid cell sub-clusters. **D** Featureplot of the feature genes of various myeloid cell sub-clusters. **E** The distribution of the cell clusters in various cancer types. **F** The phagosome signature score of myeloid cells determined by the AddModuleScore function. **G** The lysosome signature score of myeloid cells determined by the AddModuleScore function. **H** The expression profile of phagocytosis-related molecules in myeloid cells. **I** SIRPA expression level in SPP1 + macrophages (Macro-SPP1) compared with SPP1- macrophages (other macrophages). **J** Representative in situ mIHC staining images of CD68, SPP1, and SIRPα in HCC, COAD, NSCLC, and STAD samples. **K** The proportion of SIRPα-expressing macrophages in SPP1 + macrophages and SPP1- macrophages in HCC (n = 77), COAD (n = 81), NSCLC (n = 58), and STAD samples (n = 75). Statistical significance was performed by two-sided Wilcoxon tests

To further investigate the phagocytic ability of SPP1 + macrophages in various cancer types in addition to ESCC, we extracted and integrated myeloid cells from scRNA-seq data of ESCC, HCC, COAD, NSCLC, STAD, and PDAC (Fig. 2A). A total of 56,240 myeloid cells were identified and divided into 12 sub-clusters (Fig. 2B and C). Macro-SPP1 was identified by high levels of *APOE*, *SPP1,* and *TREM2*, suggesting these were lipid-associated macrophages. Macro-FOLR2 highly expressed *FOLR2*, *PLTP*, and *LYVE1* genes, indicating tissue-resident macrophages. Macro-NLRP3 highly expressed inflammation-related genes, such as *EREG, IL1B,* and *NLRP3.* Macro-CCL18 highly expressed *CCL18, APOC1*, and *PLA2G7.* Macro-MKI67 was marked with high expression of proliferation-related genes, such as *MKI67* and *STMN1* genes, suggesting a proliferative state. Similarly, Macro-MKI67 selectively expressed feature genes of Macro-SPP1 and whether Macro-MKI67 reflects a transition state of Macro-SPP1 remains unknown [36]. Monocytes were identified as Mono-CD14 (*S100A8, S100A9*, and *FCN1*) and Mono-CD16 (*LST1* and *LILRB2*). The DC cells were divided into DC-CD1C (*CD1C, CLEC10A*, and *CD1E*), DC-LAMP3 (*CCL19, CCL22*, and *FSCN1*), DC-CLEC9A (*CLEC9A* and *HLA-DPA1*), and pDC (*LILRA4, GZMB*, and *JCHAIN*). Mast cells were characterized by high expression of *TPSAB1, TPSB2*, and *CPA3* (Fig. 2C and D)*.* The feature genes of the myeloid cell sub-clusters are presented in Table S6, Additional file 2. Of note, mast cells were only identified in ESCC and COAD, while other sub-clusters were present in all six cancer types (Fig. 2E).

Consistently, macrophages exhibited a higher phagocytic score compared with DC cells and monocytes, with Macro-SPP1 demonstrating the highest phagocytosis signature score among all macrophages (Fig. 2F and G). To validate the phagocytic ability of SPP1 + macrophages in vivo, we reanalyzed scRNA-seq data from a previously published mouse tumor model. The transduction of Kras^FSF−G12D^; Rosa26^FSF-LSL−tdTomato(ai65)^; Sftpc^CreER^ (KTai65; SpcCreER) mice with a lentiviral vector containing an inverted FLPo flanked by pairs of heterotypic loxP sites enables tamoxifen-induced expression of oncogenic KRAS specifically in type II alveolar cells. Nine months after tumor initiation, KTai65; SpcCreER mice developed numerous tdTomato-positive lung tumors [27]. The micro-dissected tumor and uninvolved tissue from KTai65; SpcCreER tumor-bearing mice, as well as lung tissue from tumor-naive mice, were subjected to scRNA-seq on sorted tdTom^pos^ and tdTom^neg^ myeloid cells (enriched for myeloid cells; CD45^pos^ CD11b^pos^ and/or CD11c^pos^) and CD45^neg^ EPCAM^pos^ non-immune cells (Fig. S2A). We collected the CD45^pos^ CD11b^pos^ and/or CD11c^pos^ myeloid cells and categorized them into macrophages, DCs, monocytes, neutrophils, and eosinophils (Fig. S2B, and S2G). The tdTom^pos^ myeloid cells were mainly detected in the SPP1 + macrophage and Chil3 + alveolar macrophage sub-clusters (Fig. S2D and S2E). In tumor tissues, SPP1 + macrophages possessed the highest proportion of tdTom^pos^ cell, suggesting that these tumoral SPP1 + macrophages had the strongest phagocytic ability (Fig. S2H). Consistently, the expression pattern of *SIRPA* correlated with the phagocytic ability of myeloid sub-clusters compared to other phagocytosis-related molecules (Fig. 2H). SPP1 + macrophages also showed higher expression of *SIRPA* expression levels compared to SPP1- macrophages across six cancer types (Fig. 2I), which was verified by subsequent mIHC assays (Fig. 2J and K). Besides, the tdTom^pos^ Spp1 + macrophages in mice lung tumors exhibited high expression levels of Sirpα, aligning with our findings in humans (Fig. S2I). Collectively, these results indicated that SPP1 + macrophages possess vigorous phagocytic activity and are characterized by high SIRPα expression.

### SIRPα expression delineates subsets of SPP1 + macrophages with different functions

**Fig. 3:**
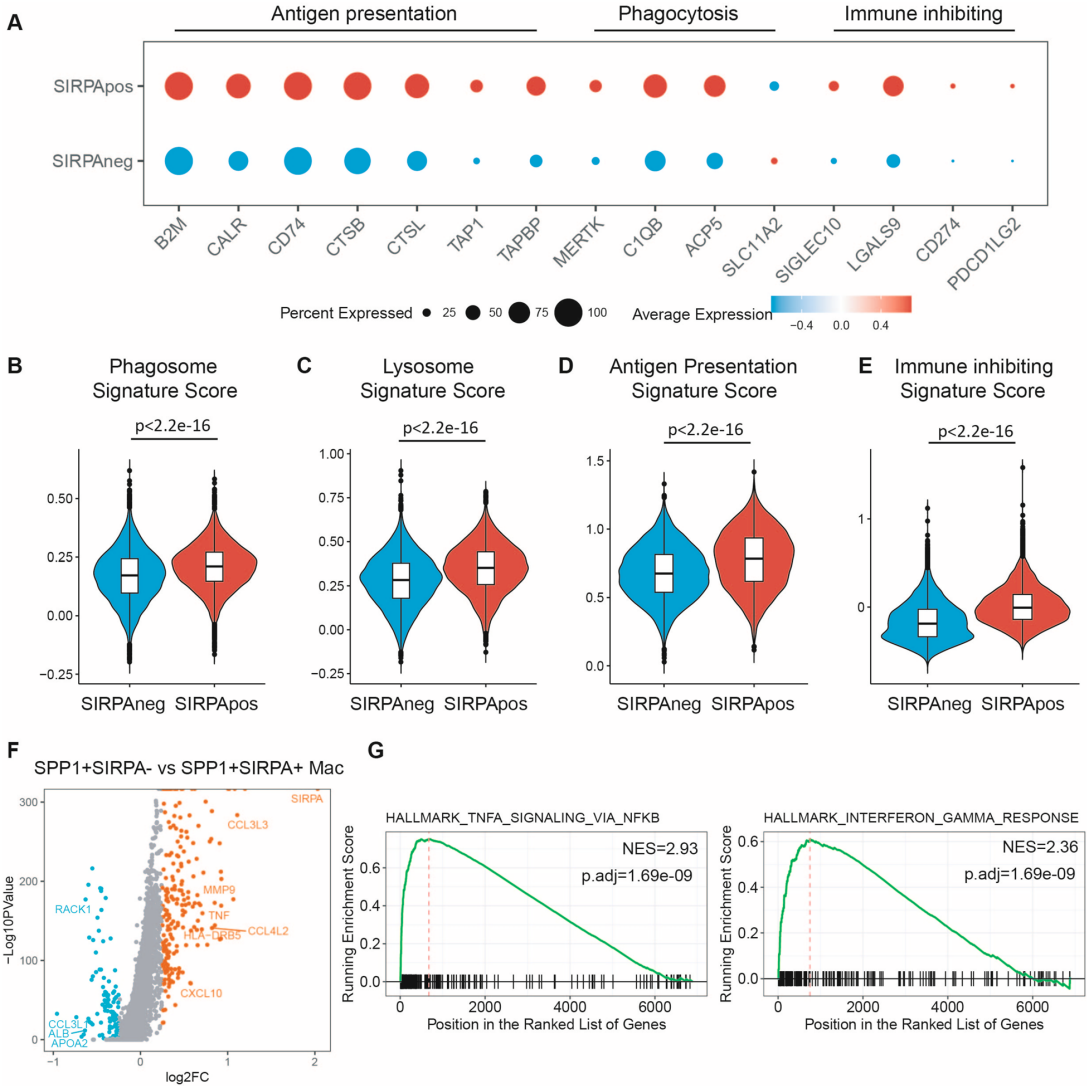
SIRPα expression delineates subsets of SPP1 + macrophages with different functions. **A** The expression profile of representative genes in the antigen presentation process, phagocytosis pathway, and immune inhibition in SPP1 + SIRPA + macrophages and SPP1 + SIRPA- macrophages. **B** The phagosome activity of SPP1 + SIRPA + macrophages and SPP1 + SIRPA- macrophages. Statistical significance was assessed using the unpaired Student’s *t*-test. **C** The lysosome activity of SPP1 + SIRPA + macrophages and SPP1 + SIRPA- macrophages. Statistical significance was assessed using the unpaired Student’s *t*-test. **D** The antigen presentation activity of SPP1 + SIRPA + macrophages and SPP1 + SIRPA-macrophages. Statistical significance was assessed using the unpaired Student’ *t*-test. **E** The immune suppressive capacity of SPP1 + SIRPA + macrophages and SPP1 + SIRPA- macrophages. Statistical significance was assessed using the unpaired Student’ s *t*-test. **F** A volcano plot showing the differential gene expression between SPP1 + SIRPA + macrophages and SPP1 + SIRPA- macrophages. **G** GSEA shows the significantly upregulated pathways in SPP1 + SIRPA + macrophages compared with SPP1 + SIRPA- macrophages

Next, to explore the impact of SIRPα on the functionality of SPP1 + macrophages, we compared SPP1 + SIRPA + macrophages with SPP1 + SIRPA- macrophages across the six abovementioned cancer types. We found that, within Macro-SPP1, SIRPA-positive cells presented a more potent phagocytic potential than SIRPA-negative cells, aligning with our earlier findings (Fig. 3A–C). Moreover, this observation was consistent across various cancer types including ESCC, COAD, STAD, HCC, PDAC, and NSCLC (Fig. S3A-F). Additionally, SIRPA-positive Macro-SPP1 exhibited an enhanced signature score of antigen presentation and immune inhibition (Fig. 3A, D, E, and Fig. S3A–F). However, the antigen-presenting ability of SPP1 + SIRPA + macrophages was inferior to that of SPP1 + SIRPA − macrophages (Fig. S3F), a discrepancy that warrants further investigation. In summary, the phenotype of SIRPA-positive Macro-SPP1 was similar to the macrophages engulfing tumor cells described previously [27]. Next, we performed a differentially expressed gene analysis between Macro-SPP1 with negative and positive SIRPA expression, revealing that SPP1 + SIRPA + macrophages exhibited higher expression of *CXCL10, HLA-DRB5, CCL4L2,* and *TNF* (Fig. 3F). Gene Set Enrichment Analysis (GSEA) indicated that TNFα signaling via the NFκB pathway and the interferon gamma response pathway were enriched in Macro-SPP1 expressing *SIRPA* (Fig. 3G). A previous study has shown that IFNγ and TNFα could enhance SIRPα expression in macrophages, which is consistent with the enrichment of interferon gamma response pathway observed in SPP1 + SIRPα + macrophages observed in our data [37].

### Spatial interaction of SPP1 + SIRPα + macrophages with tumor cells and CD8 + T cells in ESCC

**Fig. 4:**
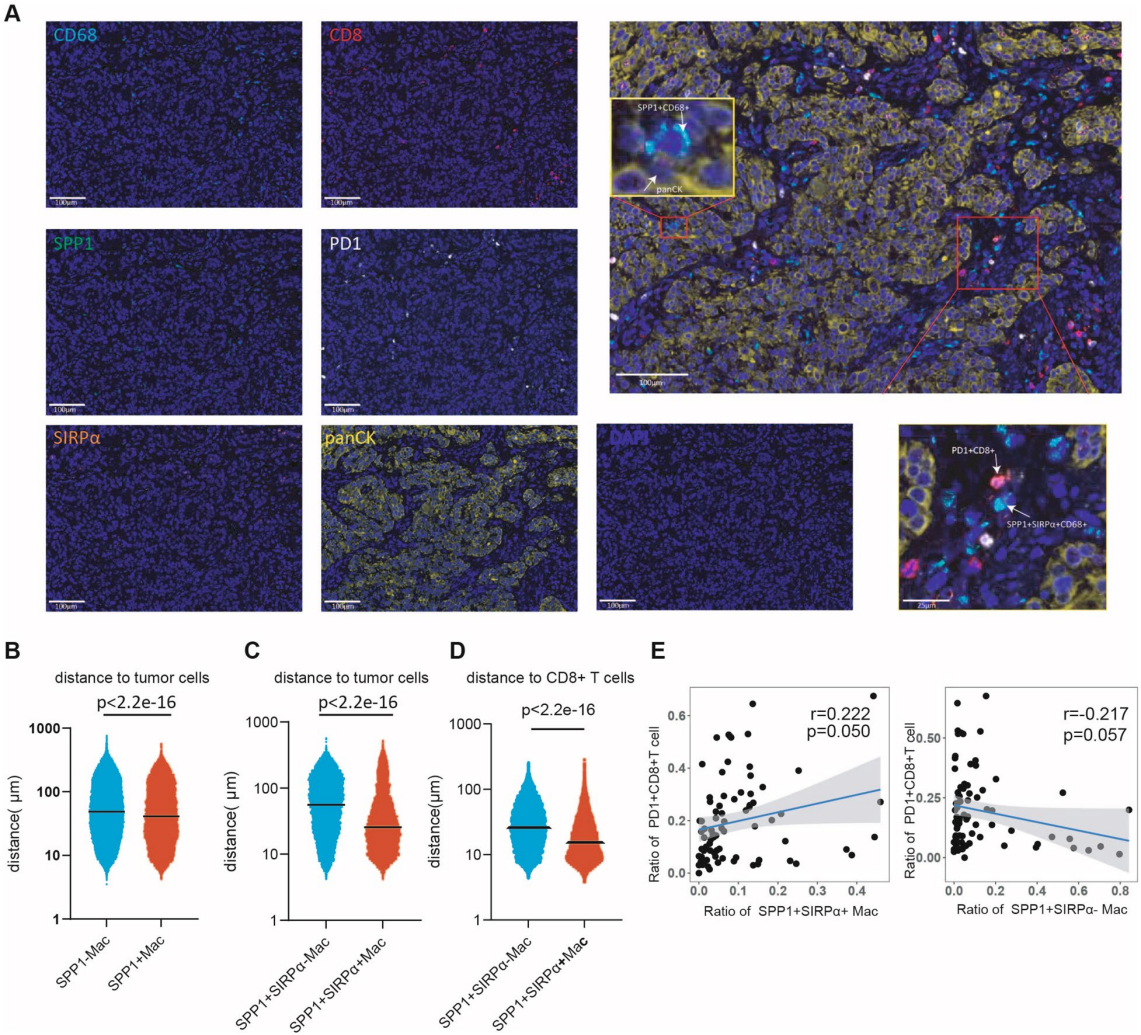
Spatial interaction of SPP1 + SIRPα + macrophages with tumor cells and CD8 + T cells in ESCC **A** Representative in situ immunofluorescent staining images of CD68, SPP1, SIRPα, panCK, PD-1, and CD8 in ESCC samples (n = 39). **B** The spatial distance analysis between SPP1 + macrophages or SPP1- macrophages and epithelial cells. Statistical significance was assessed using the Wilcox test. **C** The spatial distance analysis between SPP1 + SIRPα + macrophages or SPP1 + SIRPα- macrophages and epithelial cells. Statistical significance was assessed using the Wilcox test. **D** The spatial distance analysis between SPP1 + SIRPα + macrophages or SPP1 + SIRPα- macrophages and CD8 + T cells. Statistical significance was assessed using the Wilcox test. **E** The relationship between SPP1 + SIRPα + macrophages ratio or SPP1 + SIRPα- macrophages ratio with PD1 + CD8 + ratio in ESCC tissues. Statistical significance was assessed using Pearson’s test based on two regions per patient

The phagocytic process requires spatial proximity of phagocytes with tumor cells, and antigen-presenting or T cell suppressing by macrophages also needs spatial proximity with T cells. To investigate the spatial connection of SPP1 + SIRPα + macrophages with tumor cells and CD8 + T cells, we conducted in-situ mIHC staining in 39 ESCC patients from ESCC cohort 1 using antibodies against CD68, SIRPα, SPP1, panCK, PD-1, and CD8 (Fig. 4A)*.* The results showed that SPP1 + macrophages were in closer proximity to tumor cells than SPP1- macrophages (Fig. 4B). Moreover, within SPP1 + macrophage population, SIRPα positive cells were found to be closer to tumor cells than SIRPα negative cells (Fig. 4C). Concurrently, SPP1 + SIRPα + macrophages were also in closer proximity to CD8 + T cells than SPP1 + SIRPα + macrophages (Fig. 4D). Correlation analyses revealed that SPP1 + SIRPα + macrophages had a positive correlation with PD1 + CD8 + T cells, while SPP1 + SIRPα- macrophages had no relationship with PD1 + CD8 + T cells (Fig. 4E). The shorter spatial distance between SPP1 + SIRPα + macrophages and both tumor cells and CD8 + T cells enables their close crosstalk. Next, we performed CellChat analysis with an ESCC scRNA-seq dataset (GSE160269) to explore the specific cell interactions between SPP1 + SIRPα + macrophages and epithelial cells or CD8 + T cells. As expected, we observed increased interactions and interactional weights between SPP1 + SIRPα + macrophages and epithelial cells (Fig. S4A), among which the LGALS9-CD44 and SPP1-CD44 interactions are particularly prominent (Fig. S4B). SPP1-CD44 axis was reported as protumor interaction between macrophages and epithelial cells [38]. Similarly, we detected enhanced interactions and interactional weights between SPP1 + SIRPα + macrophages and CD8 + T cells (Fig. S4C). Of note, the MIF-(CD74 + CXCR4) interaction, LGALS9-HAVCR2 (TIM3), and SPP1-CD44 interaction were prominent between SPP1 + SIRPα + macrophages and CD8 + T cells (Fig. S4D). The SPP1-CD44 axis existing in macrophages and T cells could impair T cells function and suppress immune response [39]. Besides, the MIF-CD74 axis could suppress CD8 + T cells’ anti-tumor activity by regulating the PI3K-STAT3-PD-L1 signaling pathway, facilitating tumor proliferation and immune evasion [40].

### SPP1 + SIRPα + macrophages signature correlates with survival and clinical response to PD-1/PD-L1 inhibitors

**Fig. 5:**
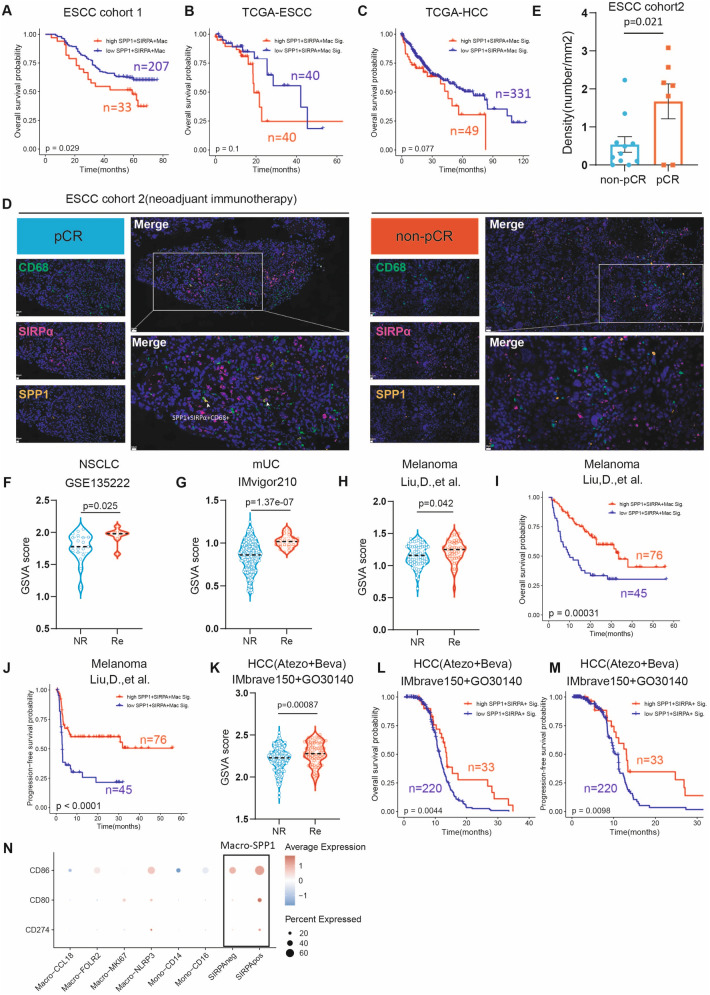
SPP1 + SIRPα + macrophages signature correlates with survival and clinical response to PD-1/PD-L1 inhibitors. A The overall survival analysis of our in-house ESCC cohort consisting of 240 patients stratified by the ratio of SPP1 + SIRPα + macrophages in macrophages. The *p*-values were calculated using a log-rank test. B The overall survival analysis in ESCC patients from TCGA stratified by the abundance of SPP1 + SIRPα + macrophages. The *p*-values were calculated using a log-rank test. **C** The overall survival analysis in HCC patients from TCGA stratified by the abundance of SPP1 + SIRPα + macrophages. The *p*-values were calculated using a log-rank test. **D** Representative in situ immunofluorescent staining of CD68, SPP1, and SIRPα in ESCC patients acquiring pCR and non-pCR when receiving neoadjuvant chemoimmunotherapy (n = 18). **E** The density of SPP1 + SIRPα + macrophages in ESCC patients acquiring pCR and non-pCR when receiving neoadjuvant immunotherapy. Statistical significance was assessed using the Student’s *t*-test. **F** The GSVA score of the SPP1 + SIRPα + macrophages signature from samples of NSCLC patients who received atezolizumab that were collected before treatment (n = 27). Statistical significance was performed by two-sided Wilcoxon tests. **G** The GSVA score of the SPP1 + SIRPα + macrophages signature from samples of mUC patients who received atezolizumab (IMvigor210) collected before immunotherapy (n = 255). Statistical significance was performed by two-sided Wilcoxon tests. **H** The GSVA score of the SPP1 + SIRPα + macrophages signature from samples of melanoma patients who received Pembrolizumab or Nivolumab treatment that were collected before treatment (n = 121). Statistical significance was performed by two-sided Wilcoxon tests. **I, J** The overall survival analysis (I) and progression-free survival analysis (J) in melanoma patients who received Pembrolizumab or Nivolumab treatment stratified by the abundance of SPP1 + SIRPα + macrophages at baseline (n = 121). The p value was calculated using a log-rank test. **K** The GSVA score of the SPP1 + SIRPα + macrophages signature in samples of HCC patients who received atezolizumab plus bevacizumab (IMbrave150 + GO30140) collected before treatment (n = 253). Statistical significance was performed by two-sided Wilcoxon tests. **L, M** The overall survival analysis (L) and progression-free survival analysis (M) in HCC patients who received atezolizumab plus bevacizumab (IMbrave150 + GO30140) stratified by the abundance of SPP1 + SIRPα + macrophages at baseline (n = 253). The *p*-values were calculated using a log-rank test. **N** The expression profiles of CD274, CD80, and CD86 across the macrophage sub-clusters.

Finally, we aimed to investigate the role of SPP1 + SIRPα + macrophages in cancer patient survival and the efficacy of immunotherapy. In our in-house ESCC cohort 1, high infiltration levels of SPP1 + SIRPα + macrophages were correlated with an unfavorable prognosis (Fig. 5A). Similarly, in both TCGA-ESCC and TCGA-HCC datasets, patients with a high SPP1 + SIRPα + macrophages signature score tended to have shorter overall survival (Fig. 5B and C). Nonetheless, given the strong antigen presenting activity and phagocytic potential of SPP1 + SIRPα + macrophages, which are crucial for adaptive immunity, we speculated that this macrophage population might play a vital role in immunotherapy. To test this, we conducted mIHC on our in-house ESCC cohort 2, comprising 18 patients who received neoadjuvant chemoimmunotherapy (Table S3, Additional file 1). We found significantly higher infiltration levels of SPP1 + SIRPα + macrophages in patients who achieved a pathologic complete response (pCR) compared to those who did not (Fig. 5D and E). We then examined the baseline abundance of SPP1 + SIRPα + macrophages in NSCLC patients [21] treated with immunotherapy and found that responders showed a higher baseline signature score of SPP1 + SIRPα + macrophages (Fig. 5F). Analysis of advanced UC patients from IMvigor210 [22] showed that responders to atezolizumab therapy also exhibited a higher SPP1 + SIRPα + macrophages signature score (Fig. 5G). In a melanoma dataset [26], we found that patients responding to Pembrolizumab or Nivolumab immunotherapy exhibited a higher signature score for SPP1 + SIRPα + macrophages than non-responders (Fig. 5H). Further survival analysis indicated that high levels of SPP1 + SIRPα + macrophages were associated with a favorable prognosis in melanoma patients treated with immunotherapy (Fig. 5I and J). HCC patients from IMbrave150 and GO30140 with a response to atezolizumab plus bevacizumab also showed a higher SPP1 + SIRPα + macrophages signature score at baseline (Fig. 5K). Survival analysis suggested that high levels of SPP1 + SIRPα + macrophages predicted a superior response to immunotherapy in HCC patients as well (Fig. 5L and M). These results suggested although tumoral enrichment of SPP1 + SIRPα + macrophages correlates with inferior survival, it implies a favorable response to immunotherapy.

**Fig. 6:**
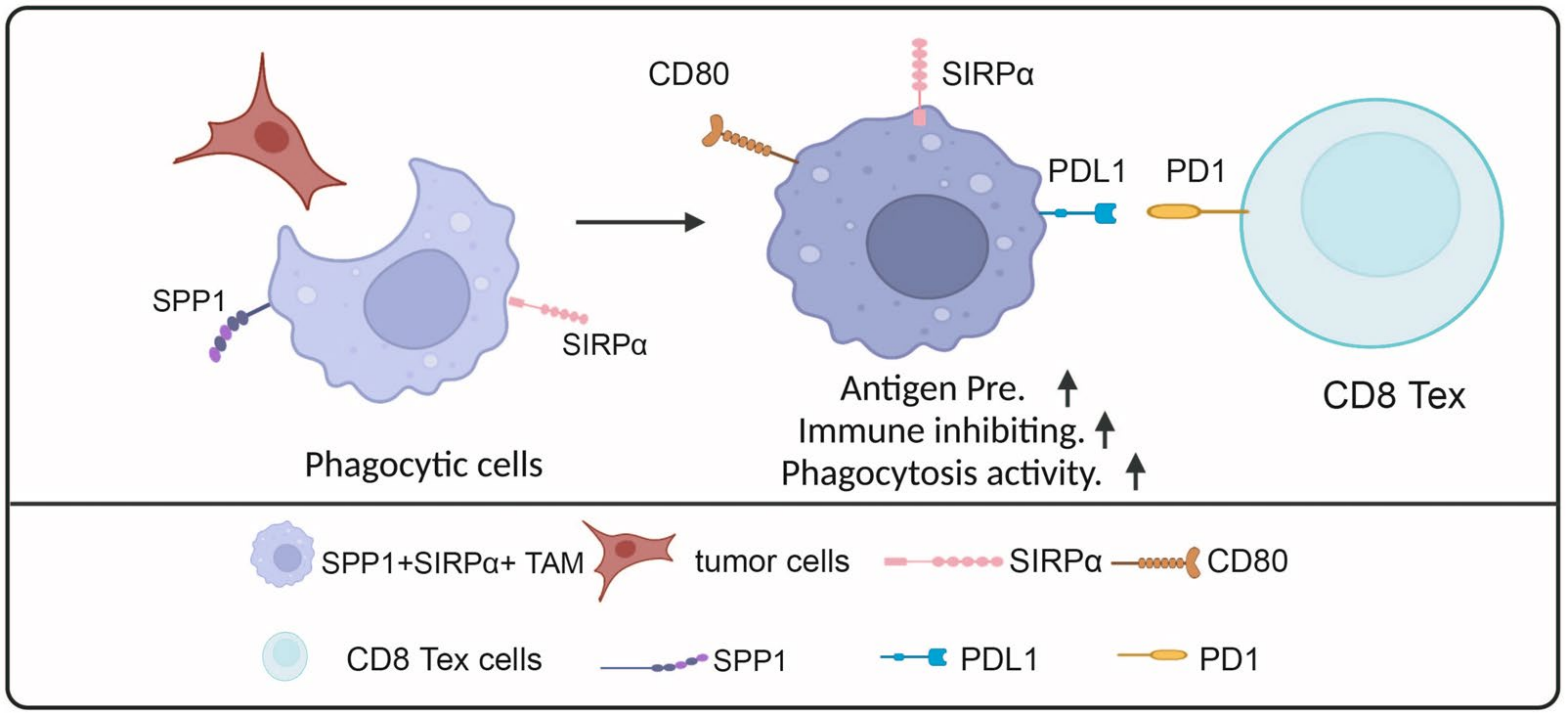
Interactions of SPP1 + SIRPα + macrophages with tumor cells and CD8 + T cells in the TME

To elucidate the underlying mechanism of SPP1 + SIRPα + macrophages in promoting the efficacy of immunotherapy, we examined the expression of the immune checkpoint *CD274*, which encodes PD-L1, and costimulatory molecules *CD80* and *CD86* across various macrophage subsets. We observed that SPP1 + SIRPα + macrophages demonstrated the highest expression levels of *CD274, CD80*, and *CD86* compared with other TAM subtypes (Fig. 5N), potentially contributing to the increased response rate to immunotherapy. The potential crosstalk among tumor cells, SPP1 + SIRPα + macrophages, and PD1 + CD8 + T cells is shown in Fig. 6.

## Discussion

SPP1 + macrophages were reported to be enriched in hypoxic and necrotic tumor regions serving as phagocytic cells for phagocytosing dying cells [6]. Macrophages that engulf tumor cells commonly exhibit distinct phenotypes. For example, macrophages that have phagocytosed neoplastic cells show upregulated expression of antigen presentation and anti-inflammatory proteins [27]. Phagocytic macrophages have been associated with enhanced antigen presentation and stronger lysosome and phagosome activity [41]. A recent study used fluorophore tdTomato-labeled neoplastic cells to identify macrophages that had phagocytosed tumor cells, and the results revealed that the engulfment of cancer cells by macrophages contributes to an immune-suppressive status of macrophages [27]. In this study, we detected a subset of SPP1 + macrophages expressing SIRPα, characterized by high lysosome and phagosome activity, T cell suppression ability, and enhanced antigen presentation ability, which is similar to macrophages engulfed tumor cells. More importantly, within SPP1 + macrophages, SIRPα positive cells exhibited enhanced phagocytic potential and were in closer proximity to both tumor cells and CD8 + T cells compared to SIRPα negative cells. The correlation of SPP1 + macrophages with exhausted CD8 + T cells has been reported previously [10]. However, our data only revealed a positive correlation between SPP1 + SIRPα + macrophages and PD1 + CD8 + T cells, with no significant correlation found between SPP1 + SIRPα- macrophages and PD1 + CD8 + T cells. These results suggest that SPP1 + macrophages expressing SIRPα represent a functional sub-population, and the heterogeneity of SPP1 + macrophages underscores the need for targeted therapies tailored to these functional subgroups [42, 43].

CD47 + tumor cells interfere with SIRPα expressed by phagocytes from being phagocytosed and sustaining suppressed immune sensing [44]. Although SIRPα was previously viewed as a negative regulator of phagocytes, our findings reveal that macrophages with robust lysosome and phagosome activity display elevated SIRPA levels. Similarly, IL4IL + phagocytic macrophages in colon and breast cancer showed up-regulated *SIRPA* expression [6]. Furthermore, a study in follicular lymphoma revealed that CD14^+^SIRPα^hi^ cells phagocytosed tumor cells more efficiently and suppressed T-cell function in vitro compared with CD14^+^ SIRPα^low^ cells [45]. These studies were consistent with our research, suggesting that the phagocytic inhibitor SIRPα reflects the phagocytic ability of SPP1 + macrophages. Thus, blocking the CD47-SIRPα interaction may strengthen the phagocytic capacity of SIRPα-positive phagocytes. SIRPα expression in macrophages also reflects other cellular phenotypes. For example, SIRPα was associated with the tumor-polarized macrophage phenotype and inflammatory state [46]. CD14^+^SIRPα^hi^ cells were characterized by their migration, differentiation, phagocytosis abilities, and suppression of T-cells [45]. Our results also indicated that SPP1 + SIRPα + macrophages possess an enhanced antigen-presenting and immuno-suppressive ability compared to SPP1 + SIRPα- macrophages. Collectively, SIRPα expression status affects the functional status and spatial distribution of SPP1 + macrophages within the TME.

Our findings showed that SPP1 + SIRPα + macrophages could be immune inhibiting and represented an unfavorable prognostic biomarker in cancer patients including ESCC and HCC. However, the abundance of SPP1 + SIRPα + macrophages at baseline positively correlated with patients’ response to immunotherapy. Expression profile analysis showed SPP1 + SIRPα + macrophages highly expressed *PD-L1*, *CD80*, and *CD86*. Tumor cells express the immune checkpoint molecule PD-L1 to escape CD8 T cell attack [47, 48]. However, recent studies revealed that PD-L1 is also expressed by immune cells, especially macrophages. In patients receiving PD-1/PD-L1 axis blockade therapy, high levels of PD-L1 expression in macrophages are associated with longer overall survival [49]. Thus, the PD-L1 expression in SPP1 + SIRPα + macrophages may partly explain why the abundance of SPP1 + SIRPα + macrophages at baseline correlated with response to PD-1/PD-L1 axis blockade immunotherapy. Additionally, SPP1 + SIRPα + macrophages possessed a strong antigen presenting signature and phagocytic potential, which is crucial for adaptive immunity. Macrophages that phagocytose tumor cells as a result of anti-CD47 antibody treatment can prime anti-tumor CD8 + T-cell responses, which indicates the role of phagocytic cells in priming the CD8 + T cell response [50–52]. SPP1 + SIRPα + macrophages as phagocytic cells might play a role in activating adaptive immunity. However, whether SPP1 + SIRPα + macrophages can directly prime CD8 + T cells warrants further investigation.

The spatial analysis showed that SPP1 + SIRPα + macrophages were in closer proximity to tumor cells compared with SPP1- macrophages. As a result, the functional status and the phenotype of SPP1 + SIRPα + macrophages are possibly influenced by the tumor cells. In fact, SPP1 + macrophages with high *MMP9* expression could be induced from THP-1 cells through their co-culture with HCC cell lines [9]. Besides, CRC cells were reported to co-localize with SPP1 + macrophages and promoted the generation of SPP1 + macrophages through HLA-G [53]. What’s more, the SPP1 + SIRPα + macrophages correlated with PD1 + CD8 + T cells positively in the TME. These results indicated the community formation of SPP1 + SIRPα + macrophages, PD1 + CD8 + T cells, and tumor cells in space, which might play a fundamental role in immune therapy.

Our study indicated that SPP1 + SIRPα + macrophages play roles in suppressing T cells and enhancing antigen presentation. Though several potential signaling pathways were identified, the specific molecular mechanisms have not been fully elucidated. Future validation assays through co-culturing SPP1 + SIRPα + macrophages with naïve or activating T cells in vitro are required. Besides, SIRPα expression could represent the phagocytic activity of SPP1 + macrophages and delineated subsets of SPP1 + macrophages with different functions. However, the specific targets and downstream signaling pathways regulated by SIRPα have not been identified.

## Conclusion

In summary, our study identified a novel subtype of SPP1 + macrophages expressing SIRPα in the TME, which possessed vigorous phagocytic ability, antigen presenting capacity, and suppressed T-cell function. Besides, the abundance of SPP1 + SIRPα + macrophages predict patients’ response to PD-1/PD-L1 inhibitors.

## Supplementary Information

Additional file 1 (This file contains the supplementary Figures, Table S1 to Table S5.)

Additional file 2 (This file contains the differently expressed feature genes of the myeloid cells in integrated scRNA-seq data. Related to Table S6.)

Additional file 3 (R code utilized in this study.)

## Data Availability

All data supporting this study are available from the corresponding author on reasonable request.

## Abbreviations

COAD: Colon adenocarcinoma
CRC: Colorectal cancer
DC: Dendritic cells
ESCC: Esophageal squamous cell carcinoma
GSVA: Gene Set Variation Analysis
GSEA: Gene Set Enrichment Analysis
HCC: Hepatocellular carcinoma
ICIs: Immune checkpoint inhibitors
KEGG: Kyoto Encyclopedia of Genes and Genomes
mIHC: Multiplex immunohistochemistry
mUC: Metastasic urothelial carcinoma
NSCLC: Non-small cell lung cancer
pCR: Pathologic complete response
PDAC: Pancreatic ductal adenocarcinoma
PMA: Phorbol 12-myristate 13-acetate
scRNA-seq: Single-cell RNA sequencing
STAD: Stomach adenocarcinoma
TCGA: The Cancer Genome Atlas Program
TMA: Tissue microarray
TME: Tumor microenvironment
