# Supplementary material for: Identification of a novel subtype of SPP1 + macrophages expressing SIRPα: implications for tumor immune evasion and treatment response prediction
Source: Exp Hematol Oncol. 2024 Dec 18;13:119. doi: 10.1186/s40164-024-00587-3 (PMC11657677; doi:10.1186/s40164-024-00587-3)
Supplement: Supplementary file 1 — Additional file 1 (This file contains the supplementary Figures, Table S1 to Table S5.) [file 40164_2024_587_MOESM1_ESM.docx]

**Supplementary Material**

**Supplementary Figures**

**
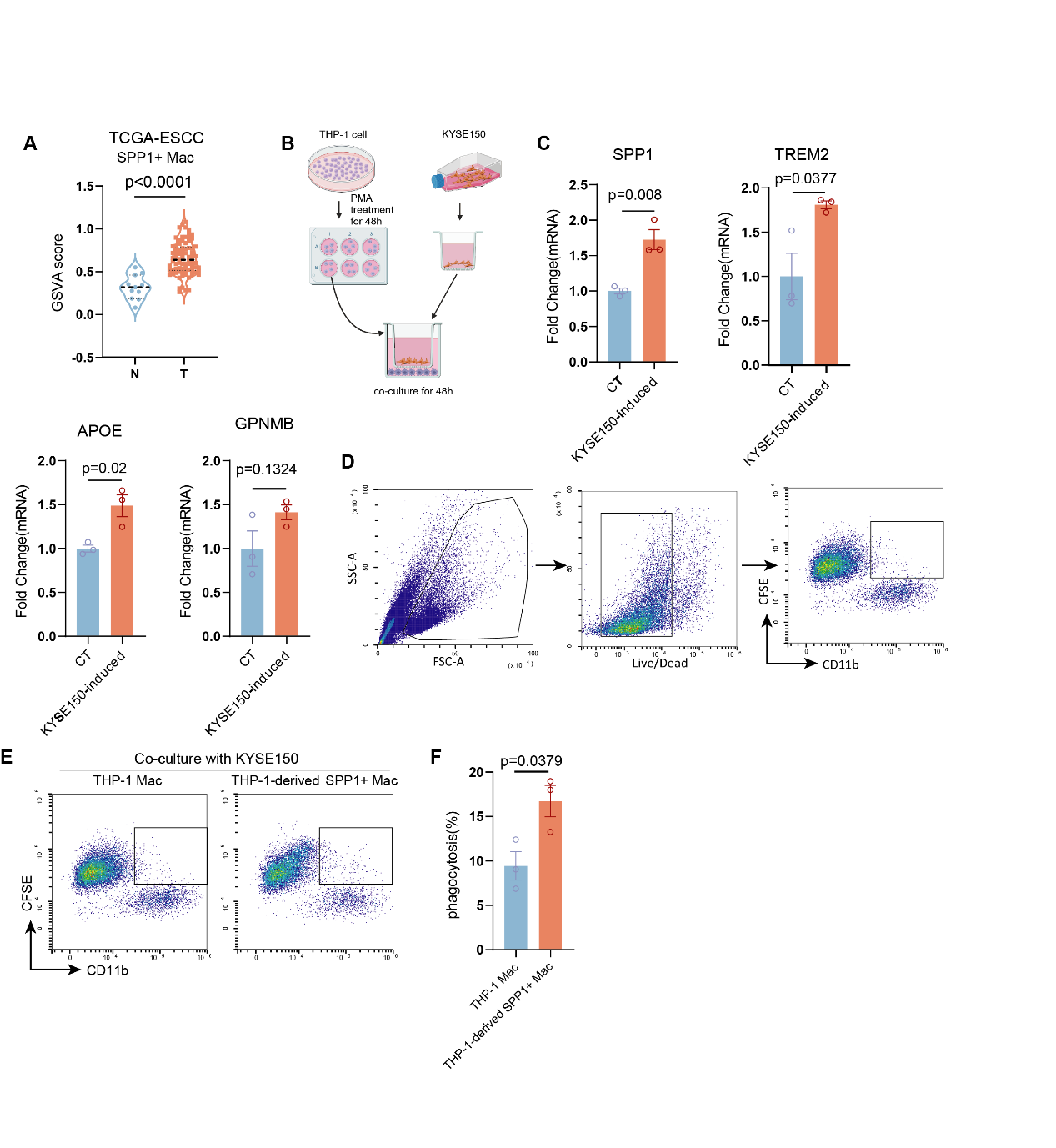
**

**Figure S1. High phagocytic activity and SIRPα expression of SPP1+ macrophages in ESCC. Related to Figure 1.**

**(A)** GSVA scores of SPP1+ macrophages in normal (N) and tumor (T) tissues in TCGA ESCC dataset (n=80). p value by two-sided Wilcoxon tests.

**(B)** The coculture procedures of THP-1 cells with ESCC cell line KYSE150 to differentiate into SPP1+macrophages-like cells.

**(C)** Validation of SPP1+ macrophages induction by RT-qPCR analysis of the feature genes expression (n=3). p value by students’ t-test.

**(D)** Flow cytometry gating strategy to evaluate the phagocytosis of SPP1+ macrophages.

**(E-F)** Phagocytic ability of THP1-derived macrophages versus THP1-derived SPP1+ macrophages (KYSE150-induced) (n=3). p values by students’ t-test.

**
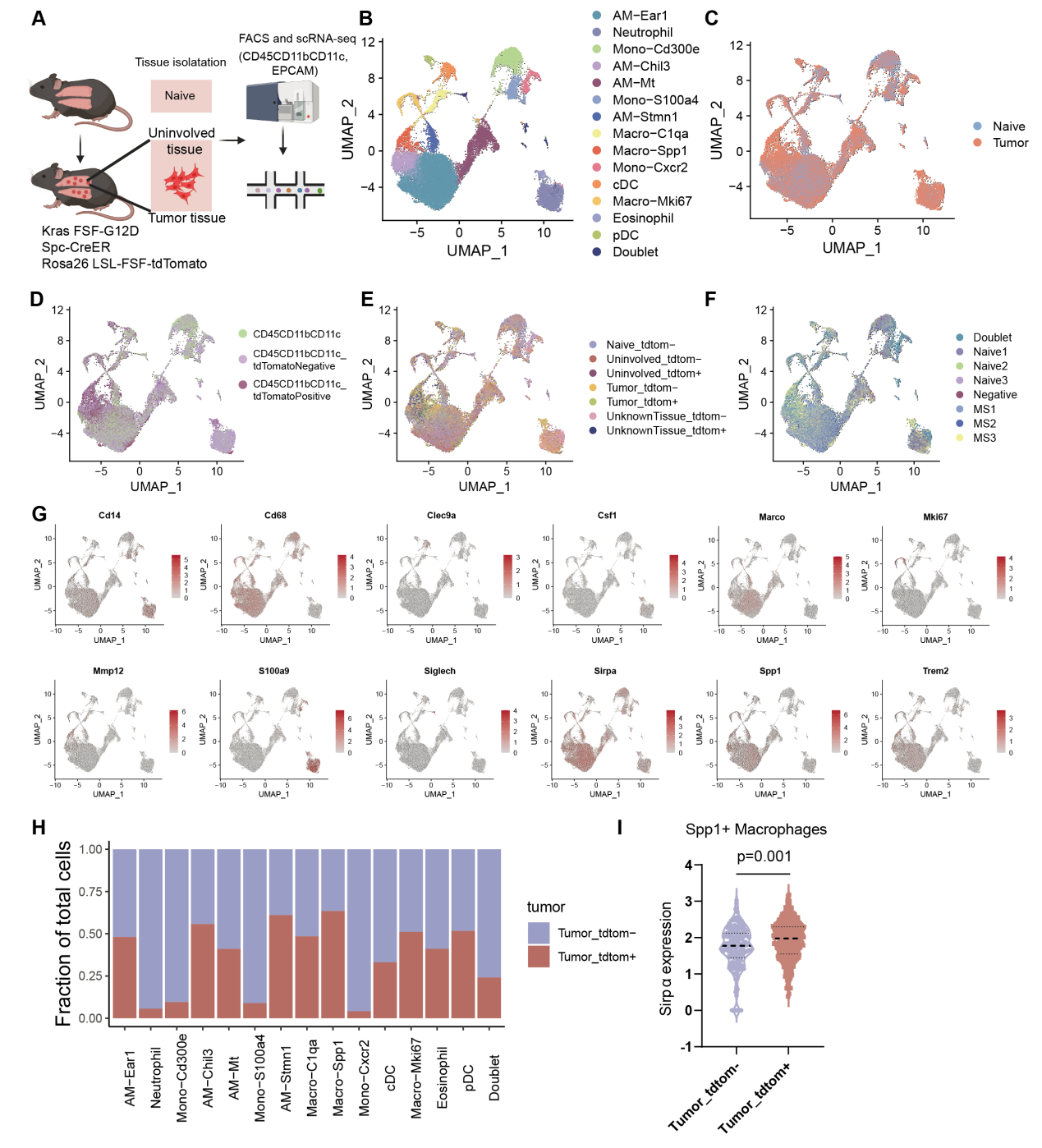
**

**Figure S2. Vigorous phagocytic activity of SPP1+ macrophages is prevalent in multiple cancer types. Related to Figure 2.**

1. The acquisition and process procedure of the scRNA-seq data from a spontaneous mice lung cancer model.
2. UMAP plot of various myeloid cell subpopulations in KTai65; SpcCreER mice that developed numerous tdTomato-positive lung tumors identified by scRNA-seq.
3. UMAP plot showing myeloid cell subclusters in naive lung tissue and tumor tissues.
4. UMAP plot showing myeloid cell subclusters tdTomato-positive or tdTomato- negative.
5. UMAP plot showing myeloid cell subclusters from different tissue origin.
6. UMAP plot showing myeloid cell subclusters from different mice.
7. UMAP plots showing marker gene expression of myeloid cell subclusters.
8. the relative tdTomato-positive and tdTomato-negative proportions of myeloid cell subclusters.
9. Sirpα expression in Spp1+ macrophages with tdTomato-positive and tdTomato-negative. p value by student’s t-test.

**
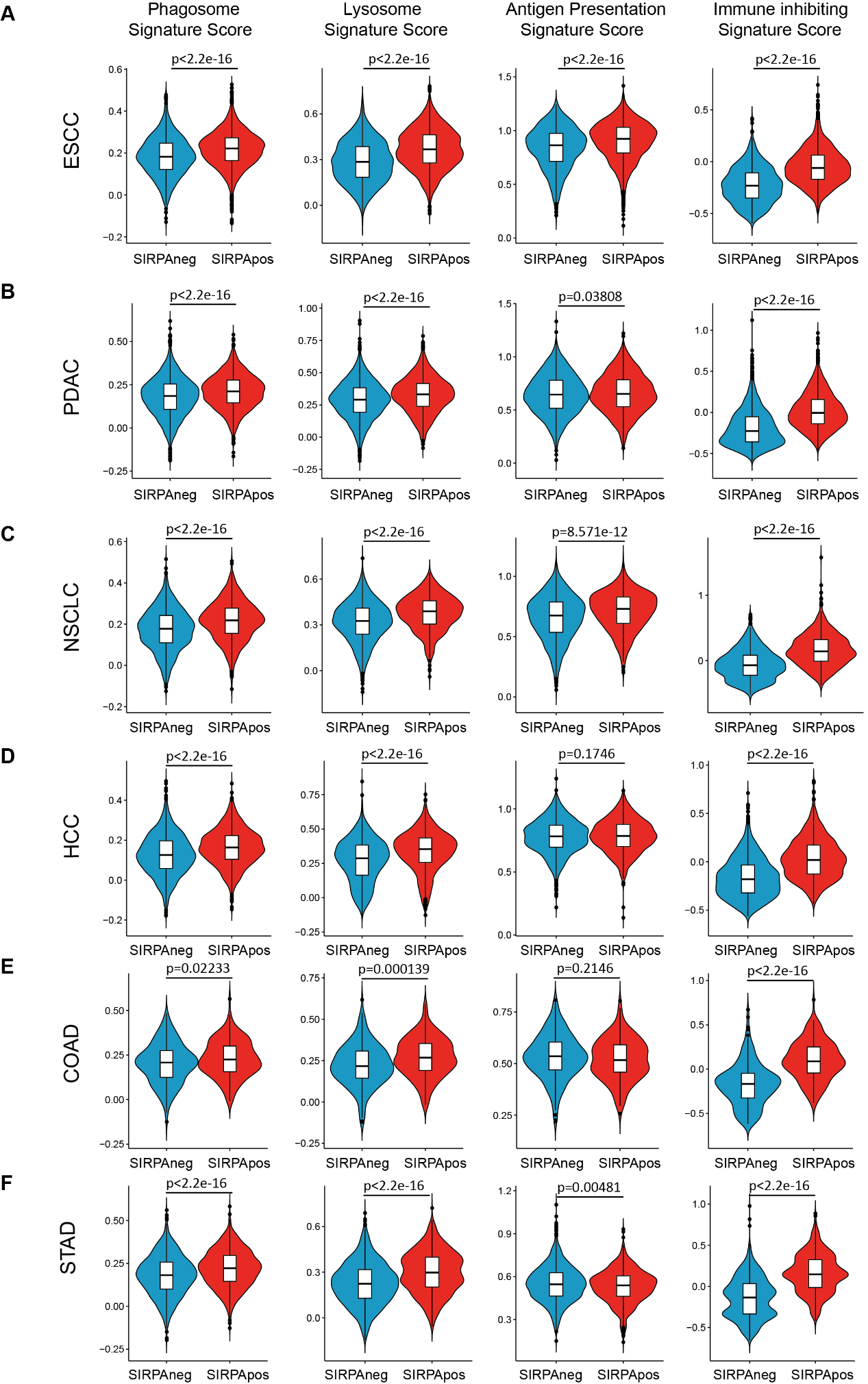
**

**Figure S3. SIRPα expression delineates subsets of SPP1+ macrophages with different function. Related to Figure 3.**

1. The phagosome activity, lysosome activity, antigen presentation activity, and immune suppressive capacity of SPP1+SIRPA+ macrophages and SPP1+SIRPA- macrophages in ESCC. Statistical significance was assessed using the unpaired Student’s t-test.
2. The phagosome activity, lysosome activity, antigen presentation activity, and immune suppressive capacity of SPP1+SIRPA+ macrophages and SPP1+SIRPA- macrophages in PDAC. Statistical significance was assessed using the unpaired Student’s t-test.
3. The phagosome activity, lysosome activity, antigen presentation activity, and immune suppressive capacity of SPP1+SIRPA+ macrophages and SPP1+SIRPA- macrophages in NSCLC. Statistical significance was assessed using the unpaired Student’s t-test.
4. The phagosome activity, lysosome activity, antigen presentation activity, and immune suppressive capacity of SPP1+SIRPA+ macrophages and SPP1+SIRPA- macrophages in HCC. Statistical significance was assessed using the unpaired Student’s t-test.
5. The phagosome activity, lysosome activity, antigen presentation activity, and immune suppressive capacity of SPP1+SIRPA+ macrophages and SPP1+SIRPA- macrophages in COAD. Statistical significance was assessed using the unpaired Student’s t-test.
6. The phagosome activity, lysosome activity, antigen presentation activity, and immune suppressive capacity of SPP1+SIRPA+ macrophages and SPP1+SIRPA- macrophages in STAD. Statistical significance was assessed using the unpaired Student’s t-test.

**
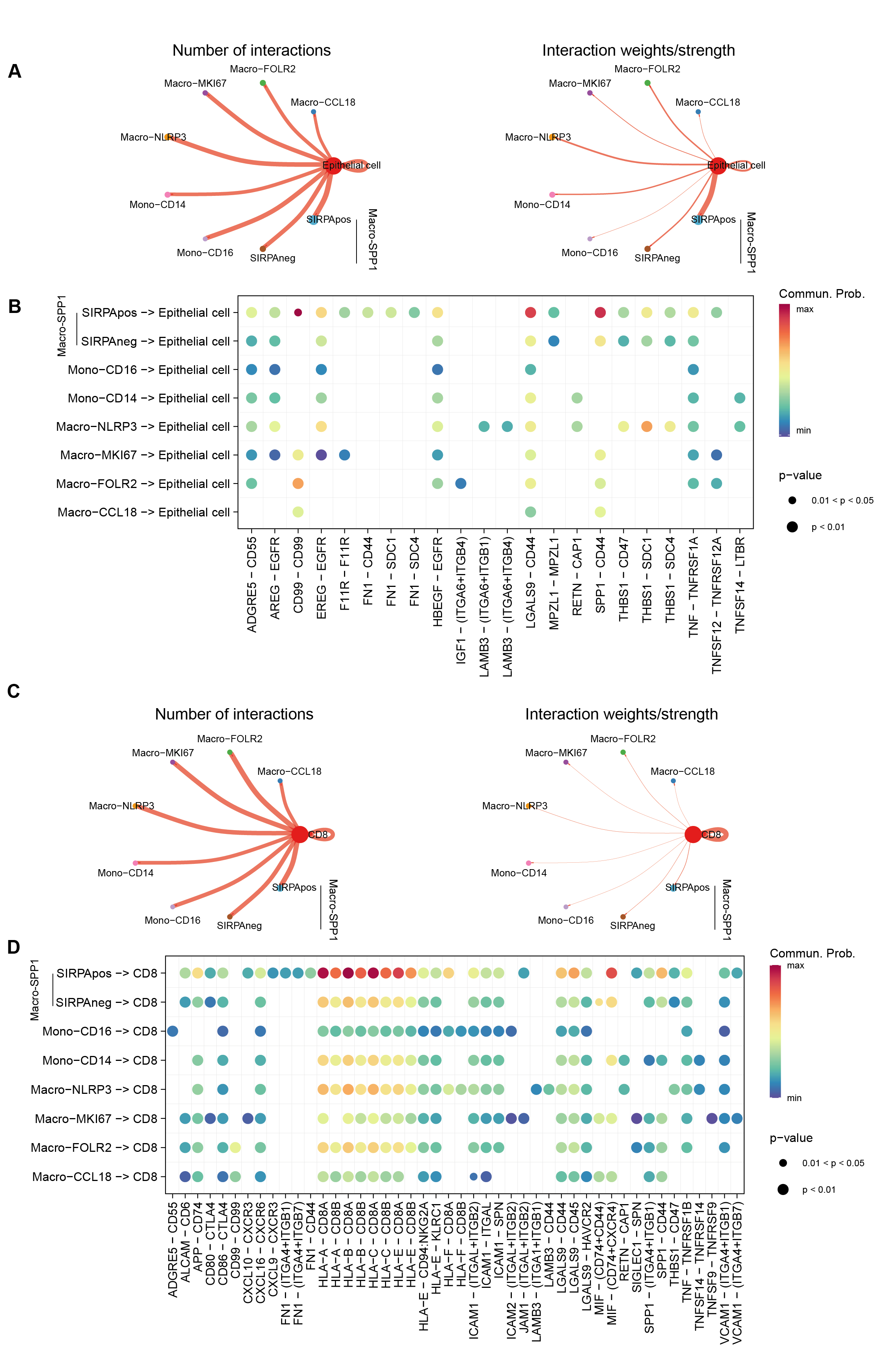
**

**Figure S4. Spatial interaction of SPP1+SIRPα+ macrophages with tumor cells and CD8+ T cells in ESCC. Related to Figure 4.**

1. Number of interactions (left) and interaction strength (right) between macrophages subsets and epithelial cells in ESCC. The edge width is proportional to the indicated number of ligand-receptor pairs (L-R pairs) (left). The edge width represents the communication probability (right).
2. The significant interactions (L-R pairs) from macrophage subsets to epithelial cells in ESCC. Dot color represents communication probabilities and dot size reflects calculated p-values. Empty space means the communication probability is zero. p-values are computed from one-sided permutation test.
3. Number of interactions (left) and interaction strength (right) between macrophages subsets and CD8+ T cells in ESCC. The edge width is proportional to the indicated number of ligand-receptor pairs (left). The edge width represents the communication probability (right).
4. The significant interactions (L-R pairs) from macrophage subsets to CD8+ T cells in ESCC. Dot color represents communication probabilities and dot size reflects calculated p-values. Empty space means the communication probability is zero. p-values are computed from one-sided permutation test.

**Supplementary Tables**

**Table S1: Patient’s features of ESCC cohort 1.**

| Variable | ESCC cohort 1(n=240) |
| --- | --- |
| **Age** |  |
| Median age (IQR), years | 60(55-64) |
| **Sex** |  |
| Male, n (%) | 199(82.9%) |
| Female, n (%) | 41(17.1%) |
| **Tobacco consumption** |  |
| No, n (%) | 75(31.3%) |
| Yes, n (%) | 158(65.8%) |
| Missing**,** n (%) | 7(2.9%) |
| **Alcohol consumption** |  |
| No, n (%) | 94(39.2%) |
| Yes, n (%) | 135(56.3%) |
| Missing**,** n (%) | 11(4.5%) |
| **Tumor location** |  |
| Upper**,** n (%) | 12(5.0%) |
| Middle**,** n (%) | 131(54.6%) |
| Lower**,** n (%) | 97(40.4%) |
| **Clinical Stage** |  |
| I, n (%) | 36(15%) |
| II, n (%) | 72(30%) |
| III, n (%) | 91(37.9%) |
| IV, n (%) | 32(13.3%) |
| Unknown, n (%) | 9(3.8%) |
| Length of tumor (IQR), cm | 3(2-4) |

Abbreviation: n.: number

**Table S2: Patient’s features of 4 cancer types for mIHC.**

| Variable | HCC cohort(n=77) | COAD cohort(n=81) | NSCLC cohort(n=58) | STAD cohort(n=75) |
| --- | --- | --- | --- | --- |
| **Age** |  |  |  |  |
| Median age (IQR), years | 49(41.5-58) | 70(60.5-79) | 61(55-67) | 57(52-69) |
| **Sex** |  |  |  |  |
| Male, n (%) | 69(89.6%) | 39(48.1%) | 55(94.8%) | 51(68%) |
| Female, n (%) | 8(10.4%) | 42(51.9%) | 3(5.2%) | 24(32%) |
| **Clinical Stage** |  |  |  |  |
| I, n (%) | 23(29.9%) | 9(11.1%) | 19(32.8%) | 16(21.3%) |
| II, n (%) | 18(23.4%) | 39(48.1%) | 14(24.1%) | 20(26.7%) |
| III, n (%) | 19(24.7%) | 28(34.6%) | 14(24.1%) | 35(46.7%) |
| IV, n (%) | 17(22.0%) | 5(6.2%) | 11(19.0%) | 4(5.3%) |
| **Size of tumor (IQR), cm** | 4.5(3.3-7.3) | / | 4(3-5) | 4(2.7-6) |
| **Metastasis, n (%)** | 14(18.2%) | 7(8.6%) | / | / |

Abbreviation: n.: number

**Table S3: Patient’s features of ESCC cohort 2.**

| Variable | ESCC cohort 2 (n=18) |
| --- | --- |
| **Age** |  |
| Median age, years | 60.5 (54-66) |
| **Sex** |  |
| Male, n (%) | 17 (94.4%) |
| Female, n (%) | 1 (5.6%) |
| **Tobacco consumption** |  |
| No, n (%) | 6 (33.3%) |
| Yes, n (%) | 12 (66.7%) |
| **Alcohol consumption** |  |
| No, n (%)  Yes, n (%) | 5 (27.8%)  13 (72.2%) |
| **Tumor location** |  |
| Upper, n (%) | 3 (16.7%) |
| Middle, n (%) | 9 (50%) |
| Lower, n (%) | 6 (33.3%) |
| **Clinical stage** |  |
| II, n (%) | 1 (5.6%) |
| III, n (%) | 8 (44.4%) |
| IV, n (%) | 9 (50 %) |
| **Treatment, n (%)** |  |
| Cisplatin, Paclitaxel plus PD-1 inhibitor | 13 (72.2%) |
| Carboplatin, Paclitaxel plus PD-1 inhibitor | 5 (27.8%) |
| **Response** |  |
| pCR, n (%) | 7 (38.9%) |
| non-pCR, n (%) | 11 (61.1%) |

Abbreviation: n.: number; pCR: pathologic complete response.

**Table S4: Primers used in this study**

|  | **Forword (5’-3’)** | **Reverse (5’-3’)** |
| --- | --- | --- |
| GPNMB | AAGTGAAAGATGTGTACGTGGTAACAG | TCGGATGAATTTCGATCGTTCT |
| TREM2 | ACTACTCTGCCTGAACAC | GCTAAATATGACAGTCTTGGA |
| SPP1 | GGGGGTCACTGCAATTAGACT | CTGTGGGGCTAGGAGATTCTG |
| APOE | CACCGGCTTTTGGGATTACCTGCGC | AAACCGCGCAGGTAATCCCAAAAGCC |
| GAPDH | GTCTCCTCTGACTTCAACAGCG | ACCACCCTGTTGCTGTAGCCAA |

**Table S5: Gene lists of immune signatures, and SPP1+ SIRPα+ macrophages signature**

| **Gene sets** | **Gene list** |
| --- | --- |
| **Phagosome** | "PIK3C3","FAB1","C1R","CTSL","CTSS","ATPeV1H","ATP6A","ATP6B","ATP6C","ATP6D","ATP6E","ATP6F","ATP6G","ATP6H","ATP6N","ATP6L","ATP6S14","ATP6Sl","C3","MBL","CD14","RAC1","THBS2S","ABCB2","ABCB3","ACTB_G1","ITGB1","ITGB2","ITGAM","CD36","FCGR3","FCGR2A","ITGA2","ITGA5","ITGAV","ITGB3","FCGR1A","TFRC","FCAR","CD107","MSR1","MRC","CD209","ITGB5","SEC61G","IGH","TUBA","TUBB","RAB5A","RAB5B","RAB7A","RAB7B","RAB5C","CYBA","NCF1","NCF2","NCF4","CANX","CALR","STX7","STX18","SEC22","OLR1","SEC61B","COLEC12","COLEC11","SFTPA","SFTPD","CLEC7A","M6PR","TLR2","TLR4","TLR6","DYNC1H","DYNC2H","DYNC1I","DYNC1LI","MPO","SEC61A","HGS","EEA1","FCGR2B","NOS1","VAMP3","STX12","CORO1A","RILP","MARCO","SCARB1","FCGR2C","THBS1","NOX2" |
| **Lysosome** | "LIPA","PPT","NAGPA","GALNS","ARSA","ARSB","IDS","GNS","DNASE2","NEU1","GLA","MANBA","GUSB","GBA","GALC","NAGA","NAGLU","FUCA","IDUA","CTSC","TPP1","CTSB","CTSG","CTSL","CTSS","CTSH","CTSK","CTSF","CTS0","LGMN","CTSD","CTSE","LGMN","AGA","SGSH","ATPeV1H","ATP6D","ATP6N","ATP6L","ATP6F","ATP6Sl","CLTA","CLTB","CLTC","MCOLN1","ABCA2","ABCB9","LYPLA3","CD63","CD68","CD107","CD164","LAMP3","IGF2R","GNPTAB","NAPSA","CTSZ","CTSW","CTSP","CTSM","GNPTG","M6PR","HGSNAT","SLC17A5","ENTPD4","MSFD8","GLB1","MAN2B1","GAA","SLC11A1","ASAH1","SMPD1","HEXA_B","ARSG","PSAP","GM2A","LIMP2","NPC1","CTNS","LAPTM","SORT1","BTS","CLN5","AP1G1","AP1B1","AP1M","AP1S1_2","AP1S3","AP3D","AP3B","AP3M","AP3S","AP4E1","AP4B1", "AP4M1","AP4S1","GGA","CTSA","NPC2","SUMF1","ACP5","ACP2","LITAF","SLC11A2","DMXL","WDR7","NCOA7" |
| **Immune Inhibiting** | "CD274","PDCD1LG2","LGALS9","SIGLEC10","VSIR","VSIG4","SIRPA" |
| **Antigen presentation** | "B2M","CALR","CANX","CD4","CD74","CD8A","CD8B","CIITA","CREB1","CTSB","CTSL","CTSS","HLA-A","HLA-B","HLA-C","HLA-DMA","HLA-DMB","HLA-DOA","HLA-DOB","HLA-DPA1","HLA-DPB1","HLA-DQA1","HLA-DQA2","HLA-DQB1","HLA-DRA","HLA-DRB1","HLA-DRB3","HLA-DRB4","HLA-DRB5","HLA-E","HLA-F","HLA-G","HSP90AA1","HSP90AB1","HSPA1A","HSPA1B","HSPA1L","HSPA2","HSPA4",'HSPA5','HSPA6','HSPA8',"IFI30","IFNA1","IFNA10",'IFNA13',"IFNA14",'IFNA16',"IFNA17","IFNA2","IFNA21","IFNA4",'IFNA5',"IFNA6","IFNA7","IFNA8","KIR2DL1","KIR2DL2","KIR2DL3","KIR2DL4","KIR2DL5A","KIR2DS1","KIR2DS3","KIR2DS4","KIR2DS5","KIR3DL1","KIR3DL2","KIR3DL3","KLRC1","KLRC2","KLRC3","KLRC4","KLRD1","LGMN","LTA","NFYA","NFYB","NFYC","PDIA3","PSME1","PSME2","PSME3","RFX5","RFXANK","RFXAP","TAP1","TAP2","TAPBP" |
| **SPP1+SIRPα+ macrophages** | "MMP12","CXCL10","C1QC","SPP1","CTSD","CTSB","SIRPA","APOE","C1QB","TREM2","ACP5","MMP9","C1QA","CTSL","APOC1","IFI6","CCL4","CXCL9","CD68","RNASE1","GPNMB","IFI27","PLD3","SPOC4-APOC2","CCL2","ISG15","PLA2G7","LGMN","CTSC","FABP5" |
| **SPP1+ macrophages** | “SPP1”,“MMP12”, "APOE","CXCL10","GPNMB",”NUPR1”, "CTSD","APOC1", "C1QC","TREM2","IFI6","CTSB",“C1QB”,“RNASE1”, ”ACP5”,”C1QA”,”IFI27”,”CTSL”,”CXCL9”,”FABP5”,”FTL”,”FN1”,”MSR1”,”CCL2”,”CD81”,”PLD3”,”ISG15”,”LIPA”,”CSTB”,”CCL18” |
