# Supplementary material for: Identification of a novel subtype of SPP1 + macrophages expressing SIRPα: implications for tumor immune evasion and treatment response prediction
Source: Exp Hematol Oncol. 2024 Dec 18;13:119. doi: 10.1186/s40164-024-00587-3 (PMC11657677; doi:10.1186/s40164-024-00587-3)
Supplement: Supplementary file 3 — Additional file 3 (R code utilized in this study.) [file 40164_2024_587_MOESM3_ESM.docx]

**Supplementary codes**

**1.Clusters division and visualization**

library(Seurat)

COM.list <- SplitObject(GEO_ESCC_integ1, split.by = "source")

COM.list <- lapply(X = COM.list, FUN = function(x) {

x <- NormalizeData(x)

x <- FindVariableFeatures(x, selection.method = "vst", nfeatures = 3000)})

features <- SelectIntegrationFeatures(object.list = COM.list)

Immune.anchors <- FindIntegrationAnchors(object.list = COM.list, anchor.features = features)

Immune.combined1 <- IntegrateData(anchorset = Immune.anchors)

DefaultAssay(Immune.combined1) <- "integrated"

Immune.combined2 <- ScaleData(Immune.combined1, verbose = FALSE)

Immune.combined3 <- RunPCA(Immune.combined2, npcs = 30, verbose = FALSE)

ElbowPlot(Immune.combined3,ndims=30)

Immune.combined3 <- RunUMAP(Immune.combined3, reduction = "pca", dims = 1:30)

Immune.combined3 <- FindNeighbors(Immune.combined3, reduction = "pca", dims = 1:30)

Immune.combined3 <- FindClusters(Immune.combined3, resolution = 0.3)

DefaultAssay(Immune.combined3) <- "RNA"

GEO_ESCC_integ <- Immune.combined3

GEO_ESCC_integ <- RunUMAP(GEO_ESCC_integ1, dims = 1:30)

DimPlot(GEO_ESCC_integ, reduction = "umap", label = TRUE, repel = TRUE)

GEO_ESCC_integ_markers <- FindAllMarkers(GEO_ESCC_integ,only.pos = TRUE, min.pct = 0.25, logfc.threshold = 0.25)

1. **Gene sets signature score calculation**

library(Seurat)

lysosomegene=data.frame(gene=c("LIPA","PPT","NAGPA","GALNS","ARSA","ARSB","IDS","GNS","DNASE2","NEU1","GLA","MANBA","GUSB","GBA","GALC","NAGA","NAGLU","FUCA","IDUA","CTSC","TPP1","CTSB","CTSG","CTSL","CTSS","CTSH","CTSK","CTSF","CTS0","LGMN","CTSD","CTSE","LGMN","AGA","SGSH","ATPeV1H","ATP6D","ATP6N","ATP6L","ATP6F","ATP6Sl","CLTA","CLTB","CLTC","MCOLN1","ABCA2","ABCB9","LYPLA3","CD63","CD68","CD107","CD164","LAMP3","IGF2R","GNPTAB","NAPSA","CTSZ","CTSW","CTSP","CTSM","GNPTG","M6PR","HGSNAT","SLC17A5","ENTPD4","MSFD8","GLB1","MAN2B1","GAA","SLC11A1","ASAH1","SMPD1","HEXA_B","ARSG","PSAP","GM2A","LIMP2","NPC1","CTNS","LAPTM","SORT1","BTS","CLN5","AP1G1","AP1B1","AP1M","AP1S1_2","AP1S3","AP3D","AP3B","AP3M","AP3S","AP4E1","AP4B1", "AP4M1", "AP4S1", "GGA","CTSA","NPC2","SUMF1","ACP5","ACP2","LITAF","SLC11A2","DMXL","WDR7","NCOA7" ))

lysosomegene = as.list(lysosomegene)

GEO_ESCC_integ1 = AddModuleScore(object =GEO_ESCC_integ1,features = lysosomegene,ctrl=100,name = "lysosomegeneScore1",assay = 'RNA')

1. **GSEA**

library(clusterProfiler)

library(enrichplot)

hallmark=read.gmt('h.all.v2022.1.Hs.symbols.gmt')

alldiff = res1[order(res1$log2FoldChange,decreasing = T),] # res1: the differently expressed genes

genelist = alldiff$log2FoldChange

names(genelist) = alldiff$gene

gsea.spp1= GSEA(genelist,TERM2GENE= hallmark, pvalueCutoff = 1, pAdjustMethod = 'BH')

p1 = gseaplot(gsea.spp1,geneSetID = 3,by="runningScore",title = gsea.spp1@result$Description[3])

1. **Survival analysis**

library(survival)

library(survminer)

library(openxlsx)

SKCM_NM_SPP1_clinical <- read_excel("SKCM_NM_SPP1_clinical.xlsx")

ssgseaScore_SKCM_NM_merge$OStime <- ssgseaScore_SKCM_NM_merge$OS/30

res.cut <- surv_cutpoint(ssgseaScore_SKCM_NM_merge,

time = "OStime",

event = "event",

variables = c("SIRPA"))

ssgseaScore_SKCM_NM_merge$SIRPAcutOS <- ifelse(ssgseaScore_SKCM_NM_merge$`SIRPA` > res.cut$cutpoint$cutpoint,"high","low")

fit.OS <- survfit(Surv(OStime, dead) ~ssgseaScore_SKCM_NM_merge$SIRPAcutOS, data = ssgseaScore_SKCM_NM_merge)

p1 <- ggsurvplot(fit.OS,

data = ssgseaScore_SKCM_NM_merge,

risk.table = FALSE,

pval = T,legend.title = "",pval.coord = c(0,0.01),

legend.labs = c("high SPP1+SIRPA+ sig.", "low SPP1+SIRPA+ sig."),legend = c(0.75,0.95),palette = c("red","blue"),xlab="Time(months)")

1. **Cell-cell interaction**

library(cellchat)

GSE160269_MacroCD8 <- merge(GSE160269_Macro2,GSE160269_CD8T)

cellchat <- createCellChat(object = GSE160269_MacroCD8,meta=GSE160269_MacroCD8@meta.data,group.by = "celltype")

CellChatDB.use <- subsetDB(CellChatDB, search =c("Secreted Signaling","ECM-Receptor","Cell-Cell Contact"))

cellchat@DB <- CellChatDB.use

cellchat <- subsetData(cellchat)

cellchat <- identifyOverExpressedGenes(cellchat)

cellchat <- identifyOverExpressedInteractions(cellchat)

cellchat <- projectData(cellchat, PPI.human)

cellchat <- computeCommunProb(cellchat, raw.use = TRUE, population.size = TRUE)

df.net <- subsetCommunication(cellchat, thresh = 100)

cellchatdb <- CellChatDB.use[["interaction"]][["interaction_name"]]

annotation <- CellChatDB.use[["interaction"]][["annotation"]]

p1 <- netVisual_bubble(cellchat, sources.use = c(2,3,4,5,6,7,8,9), targets.use = c(1),remove.isolate = FALSE)+coord_flip()

cellchat <- aggregateNet(cellchat)

groupSize <- as.numeric(table(cellchat@idents))

mat <- matrix(0, nrow = nrow(cellchat@net$count), ncol = ncol(cellchat@net$count), dimnames = dimnames(cellchat@net$count))

mat[1, ] <- cellchat@net$count[1, ]

p2 <- netVisual_circle(mat, vertex.weight = groupSize, weight.scale = T, arrow.width = 0.2,

arrow.size = 0.1, edge.weight.max = max(mat), title.name = rownames(mat)[1])
